# Acute Respiratory Failure in Critically Ill Patients with Interstitial Lung Disease

**DOI:** 10.1371/journal.pone.0104897

**Published:** 2014-08-12

**Authors:** Lara Zafrani, Virginie Lemiale, Nathanael Lapidus, Gwenael Lorillon, Benoît Schlemmer, Elie Azoulay

**Affiliations:** 1 Medical Intensive Care Unit, Saint-Louis University Hospital, Paris, France; 2 Biostatistics Department, Saint-Louis University Hospital, Paris, France; 3 Pulmonology Department, Saint-Louis University Hospital, Paris, France; University of Texas Health Science Center at Houston, United States of America

## Abstract

**Background:**

Patients with chronic known or unknown interstitial lung disease (ILD) may present with severe respiratory flares that require intensive management. Outcome data in these patients are scarce.

**Patients and Methods:**

Clinical and radiological features were collected in 83 patients with ILD-associated acute respiratory failure (ARF). Determinants of hospital mortality and response to corticosteroid therapy were identified by logistic regression.

**Results:**

Hospital and 1-year mortality rates were 41% and 54% respectively. Pulmonary hypertension, computed tomography (CT) fibrosis and acute kidney injury were independently associated with mortality (odds ratio (OR) 4.55; 95% confidence interval (95%CI) (1.20–17.33); OR, 7.68; (1.78–33.22) and OR 10.60; (2.25–49.97) respectively). Response to steroids was higher in patients with shorter time from hospital admission to corticosteroid therapy. Patients with fibrosis on CT had lower response to steroids (OR, 0.03; (0.005–0.21)). In mechanically ventilated patients, overdistension induced by high PEEP settings was associated with CT fibrosis and hospital mortality.

**Conclusion:**

Mortality is high in ILD-associated ARF. CT and echocardiography are valuable prognostic tools. Prompt corticosteroid therapy may improve survival.

## Background

Interstitial lung disease (ILD) is a group of disorders that occur either in association with identifiable causes (chiefly connective tissue disease, environmental exposures, and drugs) or as idiopathic conditions [1]. One complication of ILD is acute respiratory failure (ARF), which may develop as the inaugural manifestation or as an acute exacerbation of chronic ILD. ILD-associated ARF may require admission to the intensive care unit (ICU). Little is known about the clinical features and outcomes of ARF complicating ILD. Patients may meet Berlin’s criteria for acute respiratory distress syndrome (ARDS) [2], but whether they constitute a specific subset of ARDS is unclear. Most studies of ARDS excluded patients with previously diagnosed chronic ILD [3], [4], [5], and patients with ARDS inaugurating ILD were not studied separately [6]. ARF/ARDS complicating ILD may differ from other forms of ARF/ARDS regarding the response to corticosteroids and the outcome. Furthermore, ILD is responsible for an increase in lung stiffness that may increase the risk of ventilator-induced injury compared to other causes of ARF or ARDS. High positive end-expiratory pressure (PEEP) was associated with increased mortality in a retrospective cohort study of patients with ILD [7].

Ascribing inaugural ARF to ILD may be challenging. The early diagnosis of ILD is crucial to improve outcome prediction, choose optimal ventilator settings, and assess the appropriateness of specific treatments such as corticosteroids or immunosuppressants. Knowledge of outcome predictors that can be assessed early after ICU admission would help to guide the use of specific treatments. However, the low incidence of ILD-associated ARF requiring ICU admission has been a major obstacle to research into outcome predictors and treatment optimisation. In two studies, mortality was high, from 47% overall to 89.7% among patients who required invasive mechanical ventilation [7], [8]. As a result, intensivists may be reluctant to admit patients with ILD and ARF [8].

We conducted an 11-year retrospective study of patients admitted to our ICU with ILD-associated ARF. Our objectives were to describe the clinical and imaging study features, ventilator settings, and outcomes; and to identify early predictors of hospital mortality, long-term mortality, and corticosteroid responsiveness. In addition, in the mechanically ventilated patients, we evaluated correlations linking ventilator settings, computed tomography (CT) findings, and outcomes.

## Patients and Methods

We retrospectively studied patients with ILD-associated ARF admitted to the ICU of a 650-bed tertiary hospital (Saint-Louis University Hospital, Paris, France) whose pulmonology department is highly specialized in ILD. We included 114 consecutive adults with ILD admitted to our ICU between January 2002 and March 2013. ICU admission policies did not change during this period. Patients were identified retrospectively by searching the electronic ICU database. After ICU discharge, all patients were managed at our hospital, usually by a pulmonologist. The study was approved by the ethics committee of the French Society for Intensive Care (*Société de Réanimation de Langue Française*) (CE SRLF 13–37). Patient records were anonymized and de-identified prior to analysis.

We included patients with ILD who had ARF requiring high-dose oxygen therapy (>6 L/min), non-invasive ventilation (NIV) or, invasive mechanical ventilation (IMV). Patients had either previously diagnosed ILD meeting American Thoracic Society/European Respiratory Society (ATS/ERS) International Multidisciplinary Consensus Classification of the Idiopathic Interstitial Pneumonias [1], [9] or ILD diagnosed after ICU admission. ILD was either idiopathic or related to known causes (e.g., drugs, radiation, or connective tissue disease).

Patients with ILD were categorised into subtypes according to the ATS/ERS International Multidisciplinary Consensus Classification of the Idiopathic Interstitial Pneumonias [1] based on an extensive chart review by a panel composed of a radiologist, two pulmonologists, and an intensivist. A careful clinical exam and tests for connective tissue diseases have been performed routinely by intensivists and pulmonologists in charge of these patients to exclude an acute exacerbation of previously undiagnosed dermatomyositis, polymyositis, rheumatoid arthritis, primary Sjögren syndrome or systemic lupus erythematosus. Based on clinical examination, a consultation with a specialist in internal medicine and/or a rheumatologist (especially when articular manifestations were present) was obtained. We typically obtained serum muscle enzymes (eg. alanine aminotransferase, aspartate aminotransferase, creatine kinase), an antinuclear antibody test, a rheumatoid factor and an anti-Jo-1 antibody. If these tests were negative, additional tests for antibodies to RNA synthetases and signal recognition particle (anti-SRP) have been performed.

Acute exacerbations were defined based on criteria reported by Akira et al. [10] : subjective worsening of dyspnoea within the last month; new ground-glass opacities or consolidation by chest radiography or high-resolution computed tomography (CT); hypoxemia with a >10 mmHg decline in partial pressure of oxygen (PaO_2_); no evidence of lung infection by respiratory cultures or serological tests; and no clinical evidence of pulmonary embolism, congestive heart failure, or pneumothorax.

Hospital mortality was the primary outcome. We also assessed 1-year mortality rates, ICU and hospital lengths of stay, and response to corticosteroids. Responsiveness to corticosteroids was defined as an increase in the ratio of PaO_2_ over fraction of inspired oxygen (FiO_2_) ratio to more than 100 mmHg within 1 week of initiating high-dose corticosteroid therapy, as previously described [11], [12]. CT evidence of lung fibrosis was defined as the visualisation of traction bronchiectasis and/or honeycombing.

ARDS was defined using Berlin criteria [2] as ARF not fully explained by heart failure or fluid overload as judged by the treating physician, bilateral opacities consistent with pulmonary oedema by chest radiography or CT, and onset within 1 week after a known clinical insult or new/worsening respiratory symptoms. PaO_2_/FiO_2_ between 201 and 300 mmHg defined mild ARDS, 101 and 200 mmHg moderate ARDS, and PaO_2_/FiO_2_ less than or equal to 100 mmHg severe ARDS; these PaO_2_/FiO_2_ values were with a PEEP level at least of 5 cm H_2_O.

Pre-capillary pulmonary hypertension (estimated by ultrasonography) was defined by systolic pulmonary artery pressure > 35 mmHg without increased left ventricular filling pressure.

### Data collection

The data in Tables 1, 2 and 3 were abstracted from the medical records. The clinical, laboratory, and imaging data for each patient were reviewed by three intensivists, two of whom are also pulmonologists. Careful attention was given to recording mechanical ventilation settings including tidal volume (VT, adjusted for lung size estimated from predicted body weight), respiratory rate, peak airway pressure, plateau airway pressure, PEEP, and FiO_2_. We also recorded the use of prone positioning, inhaled nitric oxide, and neuromuscular blockers. Data on fluid balance, the Sequential Organ Failure Assessment (SOFA) score [13] on the first ICU day, and treatment limitation decisions were recorded.

**Table 1:** Main characteristics of the 83 study patients at ICU admission.

	Median [IQR] or N (%)
**Demographics**	
Age in years	61.7 [48.4–74.5]
Male	56 (67.5%)
Current smoker	64 (77%)
Known cardiovascular disease	43 (51.8%)
Poor chronic health status (Performance status ≥2)	31 (32.4%)
Exposure to pneumotoxic drugs	30 (36.1%)
**Clinical presentation**	
Days from respiratory symptom onset to ICU admission	11 [5]–[28]
Crackles at chest auscultation	76 (91.6%)
Shock	42 (50.6%)
Acute kidney injury	42 (50.6%)
Significant proteinuria	33 (45.2%)
Skin rash	25 (30.1%)
Arthralgia	11 (13.3%)
**ILD subgroups**	
**Toxic**	**22 (26.5%)**
Drug-induced ILD/Pneumoconiosis	14/3
Radiation pneumonitis/Hypersensitivity pneumonitis	2/3
**Connective tissue diseases**	**28 (33.7%)**
Rheumatoid arthritis	5/28
Scleroderma	6/28
Polymyositis/dermatomyositis	2/28
Primary Sjogren’s syndrome	3/28
Mixed connective tissue disease	5/28
Antisynthetase syndrome	2/28
Systemic lupus erythematosus/antiphospholipid syndrome	2/28
Miscellaneous	3/28
**Idiopathic interstitial pneumonia (acute ILD)**	**19 (22.8%)**
Cryptogenic organising pneumonia	7
Acute interstitial pneumonia	8
Lymphocytic interstitial pneumonia	2
Idiopathic acute eosinophilic pneumonia	2
**Acute exacerbation of chronic idiopathic ILD**	**17 (20.5%)**
Idiopathic pulmonary fibrosis (IPF)	9
Chronic idiopathic interstitial lung disease (other than IPF)	8
**Langerhans cell histiocytosis**	**2 (2.4%)**

Abbreviations: RV, right ventricular; ILD, interstitial lung disease.

**Table 2:** Severity, ICU management and outcomes.

	Median [IQR] or N (%)
**SOFA score at ICU admission**	5 [3.5–8]
**Criteria for Acute respiratory distress syndrome** *	60 (73.2%)
Mild/Moderate/Severe	4 (6.6%)/9 (15%)/47 (78.3%)
Pulmonary hypertension (RV dysfunction) 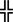	32 (40.5%)
**Ventilatory support**	
High flow oxygen	77 (92.8%)
Non-invasive ventilation alone	12 (14.5%)
Non-invasive ventilation followed by intubation	17 (20.5%)
First line invasive mechanical ventilation	33 (39.8%)
**Treatments**	
Antibiotics	78 (95.1%)
High-dose steroids	
No	29 (34.9%)
Yes, but with no respiratory response^¥^	22/54 (40.7%)
Yes, with respiratory response^¥^	32/54 (59.3%)
Other immunosuppressive drugs^£^	14 (16.9%)
**Outcomes**	
ICU length of stay	8 [3], [14]
Hospital length of stay	20 [11, 35]
Hospital mortality	34 (41%)
6-month mortality	43 (52.4%)
1-year mortality	44 (53.7%)
Treatment-limitation decisions	22 (26.5%)

*ARDS was defined using Berlin criteria [2].

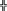
Pulmonary hypertension (right ventricular dysfunction) was assessed by transthoracic echocardiography (excluding left ventricular dysfunction).

¥ Responsiveness to corticosteroids was defined as an increase in the ratio of arterial oxygen saturation (PaO_2_) over fraction of inspired oxygen (FiO_2_) ratio to more than 100 mmHg within 1 week of initiating high-dose corticosteroid therapy.

£ cyclophophamide or rituximab.

**Table 3:** Diagnostic investigations.

	Median [IQR] or N (%)
**Chest-X-Ray**	83 (100%)
Interstitial infiltration	83 (100%)
Alveolar opacities	42 (50.6%)
Pleural effusion	17 (20.5%)
**Chest CT (n = 67, 80.7%)** *	
**Ground-glass opacities**	**47 (70.1%)**
Diffuse	43/47 (91.5%)
Focal	4/47 (8.5%)
**Fibrosis (traction bronchiectasis and/or honeycombing)**	**24 (35.8%)**
Diffuse	11/24 (45.8%)
Focal	13/24 (54.2%)
**Air-space consolidations**	**22 (32.8%)**
**Interlobular septal thickening**	**20 (29.9%)**
**Cysts**	**3 (4.5%)**
**Pleural effusions**	**20 (29.9%)**
**Pneumothorax**	**0 (0%)**
**Bronchoalveolar lavage (n = 53, 63.9%)** 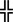	
Cell count/µL	480 [170.5–1080]
Lymphocytes >15% of total cells	20 (38.5%)
Neutrophils >10% of total cells	29 (55.8%)
Eosinophils >1% of total cells	4 (7.7%)
Diffuse intra-alveolar haemorrhage (>20% siderophages)	11 (21.2%)
**Positive bacterial culture of sputa or BAL**	10 (12.5%)
**Virus identified by PCR in BAL or nasopharyngeal aspirates**	9 (12.7%)
**Lung biopsy**	11 (13%)
**Positive auto-immune antibodies**	27 (32.5%)

*In 16 instances, CT scan was not performed because of severe hypoxemia precluding transportation to the radiological department.

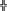
In 30 instances, results for BAL were not available either because BAL was not performed due to severe hypoxemia or the BAL results were deemed uninterpretable.

### Statistical analysis

Results are described as median and interquartile range (IQR) for quantitative variables and frequencies and percentages for qualitative variables. To determine the response to stepwise changes in PEEP, we compared the average peak and plateau airway pressures (when available), respiratory system compliance, expired VT, and PaO_2_/FiO_2_ before and after PEEP increases >4 cmH_2_O at PEEP levels >8 cmH_2_O. Multivariable Cox proportional-hazards regression was used to examine the 12-month survival mortality in patients with and without ILD-related pulmonary hypertension or CT evidence of lung fibrosis. We plotted Kaplan-Meier curves of 1-year survival for both these subgroups. We assessed the sensitivity and specificity of proteinuria, arthralgia, and skin rash for diagnosing connective tissue disease with their 95% confidence interval (exact binomial test). Possible predictors of hospital mortality and 1-year mortality were identified with the use of univariable logistic regression. Covariates available at ICU admission and associated with *p* values lower than 0.2 by univariable analysis or deemed clinically relevant were included in a multivariable logistic regression selection process. Given the number of in-hospital deaths, a maximum of four covariates was allowed in the tested models. Bootstrapping and data imputation were used to ensure the robustness of the model selection procedure: 30 datasets were generated via multiple imputations by chained equation. For each dataset, 200 bootstrap samples were drawn by random sampling with replacement. A forward/backward stepwise Bayesian information criterion (BIC)-based selection was repeated in the 6000 bootstrapped datasets [14]. The final model was the one with *p*<0.05 for all covariates selected in most bootstrap samples. We checked that omitting each of the selected variables induced no significant increase in the likelihood of hospital mortality and the model’s calibration was tested by the le Cessie–van Houwelingen goodness-of-fit test [15]. Odds ratios (ORs) and their 95% confidential intervals (CI) were calculated from the original non-imputed dataset. Statistical analysis was performed with R version 2.15.1 (R Development Core Team 2011; R Foundation for Statistical Computing, Vienna, Austria).

## Results

### Patient characteristics and outcomes

**Figure 1:**
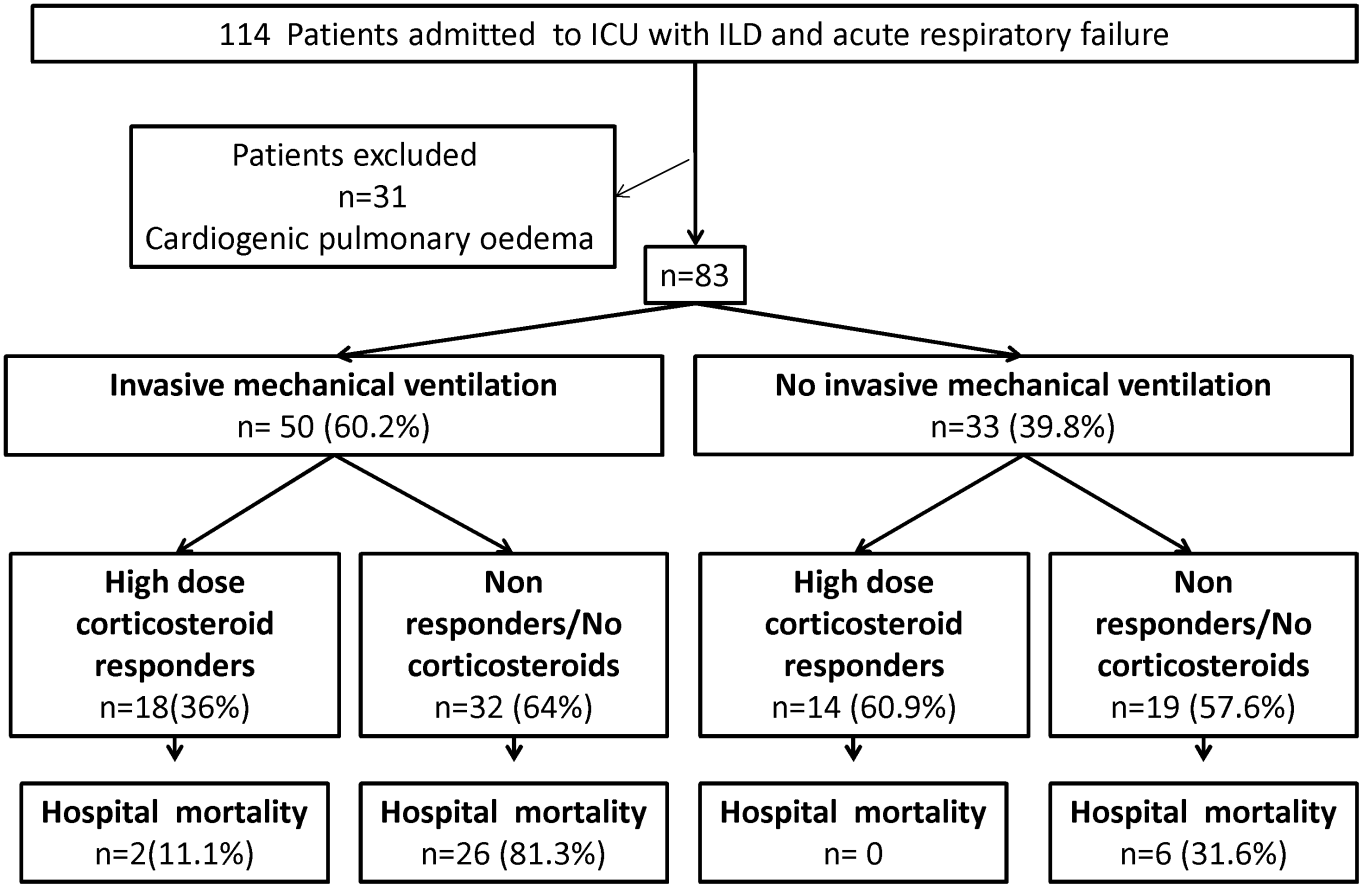
Flow chart of patients admitted to the ICU between 2002 and 2013 for acute respiratory failure and interstitial lung disease.

Of the 114 patients with ILD admitted for ARF during the 11-year study period, 31 had cardiogenic pulmonary oedema and were excluded (Figure 1), leaving 83 patients for the study. Table 1 lists their main baseline characteristics. All patients had severe hypoxemia and diffuse pulmonary infiltrates by chest radiography; none had evidence of left heart failure. Berlin criteria for ARDS were met in 60 (73%) patients, most of whom had severe ARDS (Table 2). Invasive mechanical ventilation (IMV) was required in 50 (60%) patients and vasopressors in 42 (50%) (Table 2). Acute kidney injury developed during the ICU stay in 42 (50%) patients (Table 1).

Of the 83 patients, 34 (41%) had previously diagnosed ILD and 49 (59%) had ILD diagnosed after ICU admission. Six (0.07%) patients were on low-dose supplemental oxygen prior to hospitalization. Table 1 lists the main causes of ILD. Connective tissue disease was the leading cause (33.7% of patients). A proteinuria/creatinuria ratio >30 mg/mmoL was highly sensitive for detecting connective tissue disease-associated ILD (sensitivity of 0.88, (0.69–0.97); specificity of 0.79 (0.64–0.89)). Skin rash and arthralgia were highly specific symptoms of connective tissue disease-associated ILD in ARF-related ILD (specificity of 0.98, 0.89–1.00; and of 0.84, 0.71–0.92; respectively).

Table 3 reports the results of the diagnostic investigations. CT showed ground-glass attenuation in 70% of patients and fibrosis in 35.8%. No correlations were found between CT features and broncho-alveolar lavage (BAL) fluid characteristics. Histological data were available for only 11 patients.

High-dose corticosteroid therapy was used during the ICU stay in 54 (65%) patients. Twenty-three (28%) patients required low-dose corticosteroids prior to hospitalization. Median time from hospital admission to high-dose corticosteroid therapy initiation was 5 (2–12) days. Of the 54 patients, 32 (59%) met our criteria for responsiveness. Among the 18 patients on IMV who responded to high-dose corticosteroid therapy, all but 2 were successfully extubated within a few weeks; the remaining 2 patients improved initially then experienced fatal complications. Median ICU-length of stay was 8 (3, 14) days. Hospital mortality was 41% and 1-year mortality 53.7%.

### Factors associated with hospital mortality and 1-year mortality

By univariable analysis, age, pre-capillary pulmonary hypertension, CT fibrosis, SOFA score, acute kidney injury, shock and response to high-dose corticosteroids yielded *p* values <0.005 for association with hospital mortality. Mortality rates were significantly higher in the subgroups with acute-on-chronic idiopathic ILD exacerbation or toxic ILD than in the subgroups with connective tissue disease-associated ILD or acute idiopathic interstitial pneumonia (Table 4).

**Table 4:** Determinants of hospital mortality.

	Univariate analysis		Multivariate analysis	
	OR (95%CI)	P value	OR (95%CI)	P value
**Age**	1.03 (1–1.06)	0.03		
**ILD not diagnosed previously**	0.43 (0.18–1.06)	0.07		
**ECOG Performance status ≥2**	2.01 (0.81–4.99)	0.13		
**Pre-capillary pulmonary hypertension**	5.45 (2.04–14.59)	<0.001	4.55 (1.20–17.33)	**p≤0.04**
**CT findings**				
CT micronodules	0.13 (0.02–1.12)	0.06		
CT traction bronchiectasis/honeycombing	5.82 (1.95–17.32)	0.001	7.68 (1.78–33.22)	**p≤0.01**
**Respiratory SOFA subscore**	1.96 (1.19–3.21)	0.007		
**SOFA score**	1.19 (1.05–1.35)	0.006		
**Shock**	6.70 (2.49–18.08)	<0.001		
**Acute kidney injury**	5.23 (2–13.69)	<0.001	10.60 (2.25–49.97)	**p≤0.01**
**ILD aetiology**				
Connective tissue disease	1 (ref)			
Idiopathic interstitial pneumonia (acute ILD)	0.71(0.18–2.84)	0.63		
Acute exacerbation of chronic idiopathic ILD	4.17 (1.13–15.33)	0.03		
Toxic	3.33 (1.01–10.97)	0.05		
**Ventilatory support**				
High flow oxygen	1 (ref)			
Non-invasive ventilation alone	4.75 (0.72–31.40)	0.11		
Non-invasive ventilation followed by intubation	17.4 (2.98–101.60)	<0.01		
First line invasive mechanical ventilation	10.1 (2.02–50.40)	<0.01		
**High dose corticosteroid response**				
No high dose therapy	1 (ref)			
High dose therapy with no response	34.36 (1.53–774)	<0.01		
High dose therapy with response	0.11 (0.02–0.55)	<0.01		

Abbreviations : OR, odds ratio; 95%CI, 95% confidence interval; ILD, interstitial lung disease; ECOG, Eastern Cooperative Oncology Group (the performance score can range from 0 [fully active] to 5 [dead]); CT, computed tomography of the chest; SOFA, Sequential Organ Function Assessment score.

Three factors were independently associated with hospital mortality by multivariable analysis: pre-capillary pulmonary hypertension (OR, 4.55; 95%CI (1.20–17.33)), traction bronchiectasies and/or honeycombing on CT scan (OR, 7.68; 95%CI (1.78–33.22)), and acute kidney injury (OR, 10.60; 95%CI (2.25–49.97)) (Table 4).

Multivariate analysis of variables associated with 1-year mortality showed that respiratory SOFA score (OR, 2.20; 95%CI (1.01–4.76)), performance status ≥2 (OR, 4.80; 95%CI (1.10–20.91)), traction bronchiectasies and/or honey combing (OR, 6.30; 95%CI (1.50–26.52)) and mechanical ventilation (OR, 5.18; 95%CI (1.18–22.75)) were associated with poorer survival (Table S1).

**Figure 2:**
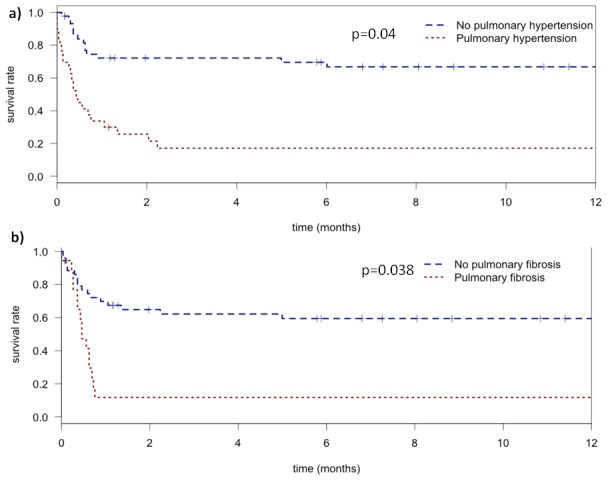
Kaplan-Meier curves of the probability of survival during 1 year after ICU admission. a) in patients with (red) and without (blue) pulmonary hypertension. b) in patients with (red) or without (blue) fibrosis (bronchiectasis/honeycombing) by CT.

After checking the proportional hazards assumption, the multivariable Cox regression identified ILD-related pulmonary hypertension and CT fibrosis as factors associated with a higher 12-month mortality: hazards ratios (HR): 3.21 (95%CI, 1.45–7.11; p = 0.004) and 2.36 (95%CI, 1.05–5.32; p = 0.038) respectively. The respective Kaplan-Meier curves are plotted figures 2a and 2b respectively for the first 12 months.

### Factors associated with responsiveness to high-dose corticosteroids

By univariable analysis, variables yielding *p* values <0.05 for the association with responsiveness to high-dose corticosteroids were age, CT fibrosis, BAL fluid lymphocytosis, and acute kidney injury. By multivariable analysis, only two factors were independently associated with non-responsiveness to high-dose corticosteroids: CT fibrosis (OR, 0.03; 95%CI (0.005–0.21)) and longer time from hospital admission to first corticosteroid bolus (OR, 0.88/day; 95%CI (0.79–0.97)) (Table 5).

**Table 5:** Univariable and multivariable analyses of factors associated with responsiveness to high-dose corticosteroids.

	Univariable analysis		Multivariable analysis	
	OR (95%CI)	*p* value	OR (95%CI)	*p* value
Age	0.94 (0.91–0.98)	0.01		
ILD not diagnosed previously	2.75 (0.85–8.90)	0.09		
ECOG performance status ≥2	0.43 (0.14–1.37)	0.15		
Long-term corticosteroid therapy	0.73 (0.23–2.32)	0.60		
Invasive mechanical ventilation	0.36 (0.11–1.20)	0.10		
Pre-capillary pulmonary hypertension	0.18 (0.05–0.62)	0.01		
CT air-space consolidation	1.29 (0.38–4.43)	0.68		
CT micronodules	6.30 (0.71–56.3)	0.10		
CT interlobular septal thickening	0.62 (0.18–2.17)	0.45		
CT ground-glass attenuation	1.50 (0.33–6.88)	0.60		
CT traction bronchiectasis and/or honeycombing	0.12 (0.03–0.44)	0.002	0.03 (0.005–0.21)	**<0.001**
CT pleural effusion	1 (0.27–3.76)	1		
BAL cellularity	1 (0.99–1)	0.42		
BAL lymphocytosis	5.78 (1.26–26.5)	0.02		
BAL polynucleosis	0.60 (0.15–2.34)	0.46		
BAL eosinophilia	1.40 (0.12–16.9)	0.79		
BAL intra-alveolar haemorrhage	0.89 (0.17–4.7)	0.89		
Respiratory SOFA subscore	0.67 (0.37–1.19)	0.17		
SOFA score	0.96 (0.82–1.12)	0.62		
Shock	0.36 (0.12–1.10)	0.08		
Acute kidney injury	0.18 (0.06–0.60)	0.01		
Time from hospital admission to first corticosteroid bolus	0.94 (0.88–1)	0.08	0.88 (0.79–0.97)	**0.001**
Aetiology				
*Connective tissue disease*	0			
*Idiopathic interstitial pneumonia (acute ILD)*	1.59 (0.26–9.54)	0.60		
*Acute exacerbation of chronic idiopathic ILD*	0.06 (0.01–0.59)	0.02		
*Toxic*	0.19 (0.05–0.82)	0.03		

Abbreviations : OR, odds ratio; 95%CI, 95% confidence interval; ILD, interstitial lung disease; ECOG, Eastern Cooperative Oncology Group (the performance score can range from 0 [fully active] to 5 [dead]); CT, computed tomography of the chest; BAL, broncho-alveolar lavage; SOFA, Sequential Organ Function Assessment score.

### Ventilator settings and effect of PEEP titration in patients given invasive mechanical ventilation –IMV)

Ventilator settings in patients managed with IMV are reported in Table S2. Prone positioning was used in only 11 patients, which precluded a valid statistical analysis of this variable.

**Figure 3:**
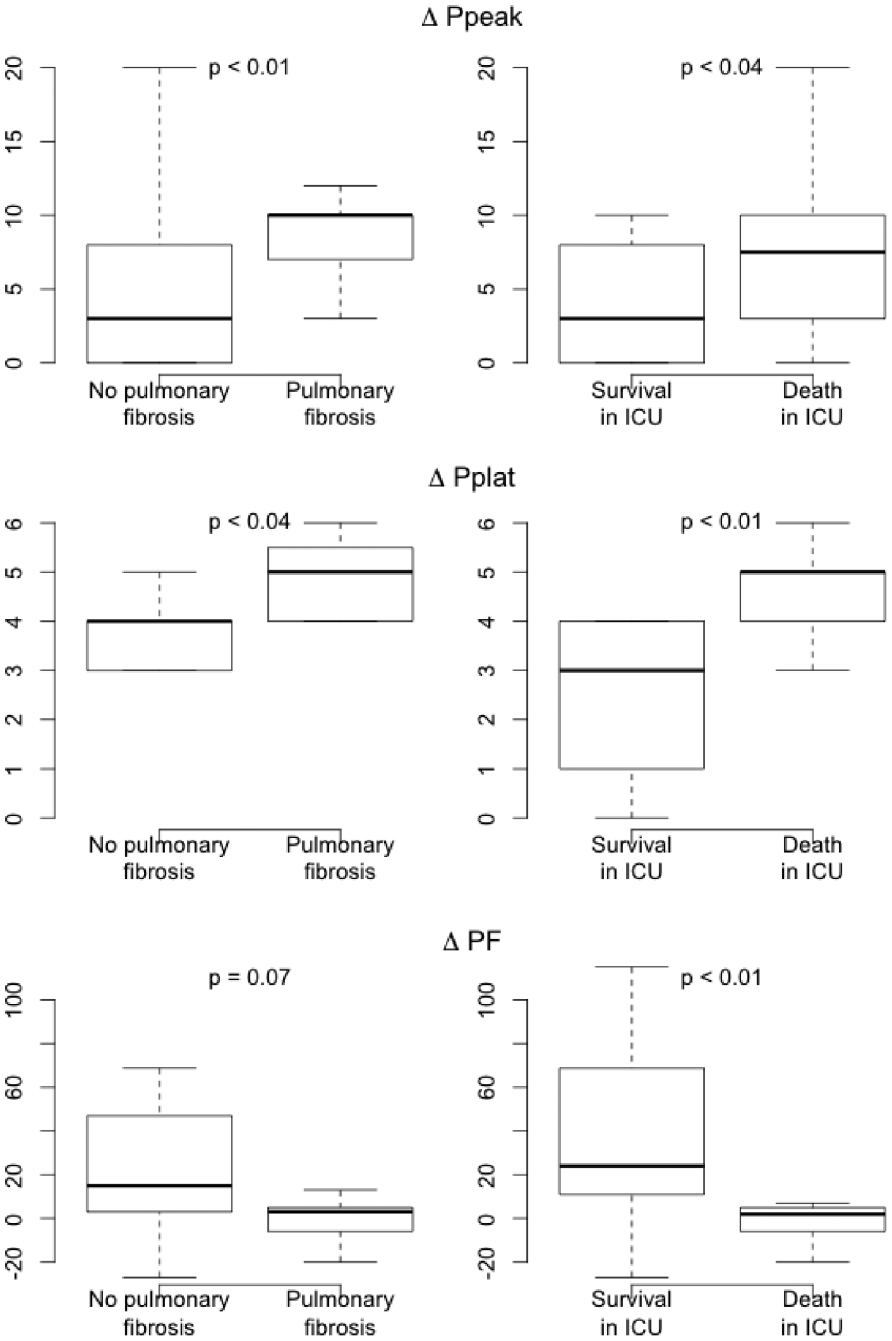
Effect of positive end-expiratory pressure (PEEP) titration in patients managed with invasive mechanical ventilation (n = 50). Right column: correlations linking variations in peak airway pressure (ΔPpeak), plateau pressure (ΔPplat), and PaO_2_/FiO_2_ (ΔPF) before and after PEEP titration to ICU mortality. Left column: correlations linking variations in peak airway pressure (ΔPpeak), plateau pressure (ΔPplat), and PaO_2_/FiO_2_ (ΔPF) before and after PEEP titration to pulmonary fibrosis by computed tomography.

PEEP titration with PEEP steps >4 cmH_2_O was performed in 37 patients during the first 24 h of mechanical ventilation. Increases in plateau pressure and peak airway pressure during PEEP titration correlated positively with ICU mortality, whereas increase in PaO_2_/FiO_2_ ratio correlated negatively with ICU mortality (Figure 3). Increases in plateau pressure and peak airway pressure during PEEP titration correlated positively with CT fibrosis (Figure 3). Of note, the maximum PEEP applied in patients with and without CT fibrosis were not different (respectively, median PEEP 11 cmH20 IQR (7.75–12) versus 10 cmH20 IQR (7.25–13.5), p = 0.81).

## Discussion

We found high hospital and 1-year mortality rates, of 41% and 53.7%, respectively, in patients admitted to our ICU for ARF complicating ILD, among whom 73% met criteria for ARDS and 60% received invasive mechanical ventilation. Fifty-nine percent of patients with ARF complicating ILD had never been previously diagnosed prior to their admission at ICU. High-dose corticosteroid therapy was administered in the ICU in two-thirds of patients, among whom 59% responded to this treatment. Fibrosis by CT was associated with hospital mortality, 1-year mortality, and failure to respond to high-dose corticosteroid therapy. In addition, acute kidney injury and pulmonary hypertension predicted hospital mortality. Delayed administration of the first corticosteroid bolus decreased the probability of obtaining a response.

Our hospital and 1-year mortality rates are consistent with a previous retrospective study of 94 patients with ILD and ARF requiring IMV in which mortality rates were 53% at hospital discharge and 59% after 1 year [7]. We did not have any information on the triage process to ICU admission and we were therefore unable to determine whether some patients with severe ILD were denied ICU admission. If such was the case, then our mortality rates would constitute an underestimation of true mortality rates in ARF complicating ILD.

More than half of our patients were not known to have ILD at ICU admission. Diagnosing ILD in patients with ARF is a major diagnostic challenge. Crackles on auscultation and pulmonary infiltrates on chest radiographs are nonspecific signs that do not separate patients with ILD from those with other forms of ARDS. A careful assessment of extra-pulmonary signs is crucial. In our study, 98% of the patients with skin lesions at ICU admission had connective tissue disease. Proteinuria was highly sensitive and a skin rash highly specific for connective tissue disease in our population of patients with ILD.

High-resolution CT is more sensitive than chest radiography for diagnosing and classifying ILD [1], [16]. We found that CT evidence of fibrosis, defined as traction bronchiectasis and/or honeycombing, was associated with hospital and 1-year mortality rates. In addition, in keeping with an earlier study [17], fibrosis by CT predicted failure to respond to high-dose corticosteroid therapy. Traction bronchiectasis and honeycombing indicate advanced histopathological alterations [18] associated with increased lung stiffness, poorer alveolar-capillary gas exchange and greater vulnerability of the lung if IMV is used.

Another predictor of hospital mortality in our cohort was pre-capillary pulmonary hypertension indicating right ventricular dysfunction at ICU admission. No patient in our cohort was treated with vasodilator or endothelial antagonist therapy specifically for pulmonary hypertension. Pulmonary hypertension in patients with ARF complicating ILD can be related to multiple factors including hypoxia, hypercapnia, mediator-induced vasoconstriction, vascular compression by oedema and fibrosis, and vascular wall remodelling. In mechanically ventilated patients, increased intra-thoracic pressures adversely influence the right ventricular afterload [19], [20], and pulmonary hypertension is of adverse prognostic significance in patients with ARDS [21] and/or idiopathic pulmonary fibrosis [22]. Whether pulmonary hypertension in ILD-associated ARF is directly involved in the occurrence of organ failures or merely reflects a more advanced stage of the lung disease remains to be elucidated.

In our cohort, BAL was performed in 64% of patients. BAL fluid cytology provides useful diagnostic information and may help to rule out active infection [23]. The treatment of ILD targets the inflammatory process, since the fibrosis is irreversible. The presence of active alveolar and interstitial inflammation suggests that high-dose corticosteroid and/or immunosuppressive treatment may prove beneficial. Lymphocytosis in the BAL fluid may indicate early-stage ILD with active inflammation. However, in our multivariable analysis BAL fluid lymphocytosis was not associated with responsiveness to high-dose corticosteroid therapy. According to current recommendations about BAL in patients with ILD overall, the BAL fluid pattern provides useful diagnostic information but does not predict the prognosis [23] or treatment response [24], [25], [26]. In our study, delayed initiation of high-dose corticosteroid therapy was independently associated with a lower response rate. This finding emphasises the need for an early diagnosis of ILD in patients admitted to the ICU with ARF.

Among our patients, 60% received IMV. Larger plateau pressure increases during PEEP titration correlated strongly with CT fibrosis and ICU mortality. In a retrospective study of ventilator settings in 53 medical and 41 postsurgical ICU patients with ILD and ARF, high PEEP was independently associated with higher mortality [7]. High PEEP failed to improve oxygenation and was associated with lung overdistension. Fibrosis results in stiffness of the lung [27], which increases the risk of ventilator-induced lung injury. In patients with ARDS and a small percentage of recruitable lung parenchyma, high PEEP was not beneficial [28]. In ILD at the stage of lung fibrosis, the proportion of recruitable lung is probably small and the risk of overdistension high, as suggested by the larger plateau pressure increases seen in patients with CT evidence of fibrosis in our study. Thus, our results indicate that caution is in order when considering the applicability of data on PEEP settings in the overall population of ARDS patients [29], [30] to patients with ILD-associated ARDS. In this last population, the use of high PEEP levels should be viewed with circumspection, particularly in patients with CT fibrosis and/or pulmonary hypertension. Similarly, recruitment manoeuvres may increase the risk of ventilator-induced lung injury in ILD-associated ARF.

Our study has several limitations. We used a single-centre retrospective design. Data on triage to ICU admission were not available, and our study may have missed patients with severe ILD who were considered too ill to benefit from ICU admission. Lung histopathological data were obtained in the ICU in only 13% of our patients, and some patients who had ILD diagnosed in the ICU may therefore have been misclassified. This source of bias was minimised in our study by a detailed review of all cases by a radiologist, two pulmonologists, and an intensivist, who used ATS guidelines [1]. The low rate of open lung biopsy probably reflects concern among intensivists about the risk of increased morbidity and mortality rates associated with this procedure. Moreover, the usefulness of urgent histopathological documentation has been challenged [31].

Our work has several important clinical implications. First, a careful physical examination for skin rash, arthralgia, and proteinuria may help to identify connective tissue disease, which is a common cause of ILD. Second, pulmonary hypertension and CT signs of fibrosis predict poor survival in all patterns of ILD with ARF. Third, whether a high lymphocyte count in BAL fluid predicts a good response to corticosteroids remains unclear. Fourth, when high-dose corticosteroid therapy is indicated, it should be started promptly to maximise the likelihood of a response. Finally, in patients managed with IMV, high PEEP levels may be harmful, most notably in patients with lung fibrosis.

## Supporting Information

Table S1 **Determinants of 1-year mortality.**
(DOC)

Table S2 **Ventilator settings in the 50 patients managed with invasive mechanical ventilation.**
(DOC)
