# Supplementary material for: Acute Respiratory Failure in Critically Ill Patients with Interstitial Lung Disease
Source: PLoS One. 2014 Aug 12;9(8):e104897. doi: 10.1371/journal.pone.0104897 (PMC4130629; doi:10.1371/journal.pone.0104897)
Supplement: Table S1 — Determinants of 1-year mortality. (DOC) [file pone.0104897.s001.doc]

**Table S1. Determinants of 1-year mortality**

|  | **Univariate analysis** | | **Multivariate analysis** | |
| --- | --- | --- | --- | --- |
|  | **OR (95%CI)** | **P value** | **OR (95%CI)** | **P value** |
| **Age** | 1.05 (1.02-1.09) | 0.001 |  |  |
| **ILD not diagnosed previously** | 0.33 (0.13-0.83) | 0.002 |  |  |
| **ECOG Performance status ≥2** | 2.56 (1.05-6.50) | 0.04 | 4.80 (1.10-20.91) | **p≤0.04** |
| **Cardiovascular disease** | 1.63 (0.68-3.98) | 0.28 |  |  |
| **Pre-capillary pulmonary hypertension** | 5.12 (1.88-13.90) | 0.001 |  |  |
| **CT findings** |  |  |  |  |
| CT micronodules | 0.21 (0.04-1.09) | 0.06 |  |  |
| CT traction bronchiectasis/honeycombing | 5.51 (1.72-17.64) | 0.004 | 6.30 (1.50-26.52) | **p≤0.02** |
| **Respiratory SOFA subscore** | 2.30 (1.39-3.82) | 0.001 | 2.20 (1.01-4.76) | **p≤0.05** |
| **SOFA score** | 1.13 (1.01-1.28) | 0.04 |  |  |
| **Invasive Mechanical ventilation** | 4.13 (1.61-10.54) | 0.003 | 5.18 (1.18-22.75) | **p≤0.03** |
| **Acute Kidney Injury** | 3 (1.22-7.39) | 0.02 |  |  |
| **ILD aetiology** |  |  |  |  |
| Connective tissue disease | 1 (ref) |  |  |  |
| Idiopathic interstitial pneumonia (acute ILD) | 0.63 (0.18-2.16) | 0.46 |  |  |
| Acute exacerbation of chronic idiopathic ILD | 3.75 (0.96-14.65) | 0.06 |  |  |
| Toxic | 2.03 (0.64-6.49) | 0.23 |  |  |

Abbreviations : OR, odds ratio; 95%CI, 95% confidence interval; ILD, interstitial lung disease; ECOG, Eastern Cooperative Oncology Group (the performance score can range from 0 [fully active] to 5 [dead]); CT, computed tomography of the chest; SOFA, Sequential Organ Function Assessment score

|  |  |  |
| --- | --- | --- |
|  |  |  |
