# Supplementary material for: Acute Respiratory Failure in Critically Ill Patients with Interstitial Lung Disease
Source: PLoS One. 2014 Aug 12;9(8):e104897. doi: 10.1371/journal.pone.0104897 (PMC4130629; doi:10.1371/journal.pone.0104897)
Supplement: Table S2 — Ventilator settings in the 50 patients managed with invasive mechanical ventilation. (DOC) [file pone.0104897.s002.doc]

Table S2. Ventilator settings in the 50 patients managed with invasive mechanical ventilation

|  | Median (IQR) |
| --- | --- |
| VT, mL/Kg PBW | 5.8 (5.4-6.9) |
| Set respiratory rate | 26 (24-30) |
| PEEP maximum, cmH_2_O | 10 (8-12) |
| Plateau pressure, cmH_2_O | 27 (24.5-28) |
| Peak airway pressure, cmH_2_O | 43 (38.5-50) |
| Static compliance  mL/kg/PBW/cm H_2_O | 26 (21.5-33.5) |
| Dynamic compliance mL/Kg/PBW/cmH_2_O | 10 (9-12.8) |
| Prone positioning (hours) (n=11) | 14 (12-16) |
| Neuromuscular blocker (n=45) |  |
| Inhaled nitric oxide (n=6) |  |

VT, tidal volume; PBW, predicted body weight; PEEP, positive end-expiratory pressure
